# Mitogenomic Characterization, Genetic Diversity, and Matrilineal Phylogenetic Insights of the Marbled Goby (*Oxyeleotris marmorata*) from Its Native Range in Indonesia

**DOI:** 10.3390/ijms27010140

**Published:** 2025-12-22

**Authors:** Sarifah Aini, Angkasa Putra, Hye-Eun Kang, Mira Maulita, Sang Van Vu, Hyun-Woo Kim, Kyoungmi Kang, Shantanu Kundu

**Affiliations:** 1 Interdisciplinary Program of Marine and Fisheries Sciences and Convergent Technology, Pukyong National University, Busan 48513, Republic of Korea; 2 Institute of Marine Life Science, Pukyong National University, Busan 48513, Republic of Korea; 3 Department of Aquatic Resources Management, Jakarta Technical University of Fisheries, Ministry of Marine Affairs and Fisheries, Jakarta 12520, Indonesia; 4 Faculty of Biology, University of Science, Vietnam National University, Hanoi 11400, Vietnam; 5 Department of Marine Biology, Pukyong National University, Busan 48513, Republic of Korea; 6 Research Center for Marine Integrated Bionics Technology, Pukyong National University, Busan 48513, Republic of Korea; 7 Marine Integrated Biomedical Technology Center, National Key Research Institutes in Universities, Pukyong National University, Busan 48513, Republic of Korea; 8 Department of Biology, Faculty of Science and Technology, Airlangga University, Surabaya 60115, Indonesia; 9 Ocean and Fisheries Development International Cooperation Institute, College of Fisheries Science, Pukyong National University, Busan 48513, Republic of Korea; 10 International Graduate Program of Fisheries Science, Pukyong National University, Busan 48513, Republic of Korea

**Keywords:** gobies, freshwater fish, mitochondrial DNA, haplotyping, phylogeny, evolution, Southeast and East Asia

## Abstract

Butidae is a family of teleost fishes with diverse morphological and ecological adaptations, including the marbled goby (*Oxyeleotris marmorata*), a large species of high economic value in Southeast and East Asia. The previous mitogenomic studies on cultured populations of *O. marmorata* from non-native habitats have provided limited insights into genetic divergence, structural variation, and evolutionary relationships. Hence, this study presented the complete mitochondrial genome of *O. marmorata* from its native habitat in Indonesia, providing structural characterization, assessment of genetic diversity, and matrilineal phylogenetic analysis. The circular mitogenome was 16,525 bp, comprising 37 genes and a non-coding control region (CR). The gene organization and strand distribution were conserved among *Oxyeleotris* species, with 28 genes on the heavy strand and nine on the light strand, and a pronounced A+T compositional bias. The comparative analyses of *O. marmorata* (from both native and cultured habitats) and *Oxyeleotris lineolata* mitogenomes revealed minor variations in intergenic spacers, gene overlaps, protein-coding gene (PCGs) lengths, and codon usage patterns. Conversely, the nonsynonymous and synonymous substitution ratios observed in species of the family Butidae and its closest related family (Eleotridae) indicate strong purifying selection in the present dataset. Notably, the ATG was the predominant start codon, whereas the COI gene utilized GTG, and amino acid composition analysis demonstrated high frequencies of arginine, leucine, and serine. Most transfer RNAs retained the canonical cloverleaf secondary structure except for trnS1, which lacked a functional dihydrouridine arm, whereas the CR contained four conserved sequence blocks with variable nucleotide motifs and no detectable tandem repeats. The haplotype analysis of native (Indonesia) and introduced populations (China) highlighted three haplotypes with high diversity (Hd = 1.0000) and substantial nucleotide variation (π = 0.6667). The genetic divergence across 13 PCGs was gene-specific, with COI and ND5 showing the highest variation, while ND4L and ATP8 were highly conserved. The phylogenetic analyses based on concatenated 13 PCGs using both Bayesian Inference and Maximum Likelihood methods revealed that *Oxyeleotris* forms a monophyletic clade and is closely related to *Bostrychus sinensis*. In addition, the broader phylogenetic framework inferred the matrilineal relationships within the family Butidae and its closest related family, Eleotridae. This study also recommends expanding analyses to include the mitogenomes of the remaining 17 *Oxyeleotris* species, together with comprehensive genomic data, to further elucidate their genetic architecture, evolutionary history, and ecological adaptability across diverse aquatic ecosystems.

## 1. Introduction

The family Butidae comprises teleost fishes within the order Gobiiformes, commonly known as sleeper gobies or gudgeons [1,2]. Initially, this group was classified as a subfamily (=Butinae) within Eleotridae, but a taxonomic revision in the fifth edition of Fishes of the World recognized Butidae as a distinct family [1]. This reclassification is corroborated by molecular phylogenetic analyses, which reveal that Butidae constitutes a sister clade to Eleotridae [3,4,5,6]. Currently, Butidae encompasses 57 valid species under 12 genera, distributed widely in tropical and subtropical freshwater and brackish habitats in Africa, Asia, Australia, and Oceania, with the highest diversity observed in Papua New Guinea, Australia, and New Zealand [2,3]. Morphologically, members of this family are characterized by a robust, cylindrical body, dorsal fins with spines and rays, pelvic fins that are not connected by a membrane, relatively thick scales, and a typical body length of 10–25 cm [7,8]. Most species are benthic due to the absence of a swim bladder, and their high tolerance to hypoxia and salinity variation makes them valuable model organisms for experimental studies in physiology and ecology [9,10,11]. Therefore, morphological diversity, behavioral strategies, and ecological adaptations within this family reflect their ability to colonize and survive across a range of aquatic habitats [12].

Within Butidae, the genus *Oxyeleotris* currently includes 19 valid species, distributed from the Sundarbans (India) and Yunnan (China) to mainland and island Southeast Asia, Australia, and Oceania [2]. While most species of Butidae are small, the marbled goby (*Oxyeleotris marmorata*) is notable as a large freshwater species, reaching up to 65 cm in length, with significant value in both aquaculture and the ornamental fish trade [13,14,15]. This species is well-known for its rapid growth, prolonged aerial survival via air breathing, and demersal predatory behavior feeding on small fish, crustaceans, and aquatic invertebrates [13,16,17]. Its natural habitats incorporate rivers, lakes, swamps, and canals, particularly within the Mekong and Chao Phraya basins, Cambodia, Thailand, Malaysia, Singapore, Indochina, Philippines, and Indonesia [2,14,18]. Outside its native range, *O. marmorata* has been introduced to China via Thailand, where it has hybridized with the native Chinese goby, *Bostrychus sinensis* [19,20]. This phenomenon likely results from close phylogenetic affinity and overlapping habitat preferences, and may have been deliberately facilitated through artificial interspecific hybridization for aquaculture purposes [19,20,21,22,23]. Although such hybridization can confer commercial benefits in aquaculture, the introduction of *O. marmorata* into non-native ecosystems may poses ecological risks, including potential threats to native goby populations and disruption of its genomic integrity [19]. Moreover, *O. marmorata* is traditionally believed to possess therapeutic properties, often consumed post-surgery or postpartum to accelerate recovery, further increasing its market demand and economic value in Southeast and East Asia [13,14,20].

Beyond ecological and economic significance, the mitochondrial genomic studies of *O. marmorata* have previously been conducted on specimens from aquaculture in China, outside their native range [19,20]. The mitogenome is a powerful molecular marker in phylogenetic and evolutionary studies due to its compact structure, high copy number, rapid mutation rate, and lack of recombination [24,25]. In vertebrates, the mitogenome typically consists of circular DNA (~16–17 kb) encoding 37 genes, including 13 protein-coding genes (PCGs), two ribosomal RNA (rRNA) genes, 22 transfer RNA (tRNA) genes, and a non-coding control region (CR) critical for replication and transcription [26,27]. Due to maternal inheritance and high resolution in distinguishing closely related species, the mitogenomic data are extensively used for species identification, population genetics, and phylogeography in many teleosts [28,29]. The comparative mitogenomic analyses have also provided key insights into genetic diversity, lineage divergence, and adaptive evolution in gobies [30,31]. However, the previous studies on *O. marmorata* have lacked in-depth insights into structural variation, genetic diversity, and detailed matrilineal phylogenetic interpretations. This highlights the need for comprehensive mitogenomic analysis of this species from its native range to understand genetic variation relative to cultured specimens and to elucidate the phylogenetic relationships. In light of these considerations, the present study aims to: (i) generate the complete mitogenome of *O. marmorata* from its native range in Indonesia; (ii) provide a detailed structural characterization and comparative assessment of mitogenomic variation among *Oxyeleotris* congeners, including *O. marmorata* from both native and non-native ranges; and (iii) evaluate the genetic diversity and matrilineal phylogenetic relationships of the family Butidae and its closest related family, Eleotridae. By integrating mitogenomic data with in-depth genetic analyses, this study provides new insights into the systematics and genetic diversity of *O. marmorata*, particularly across native and non-native populations.

## 2. Results

### 2.1. Mitogenome Structure and Organization

**Figure 1:**
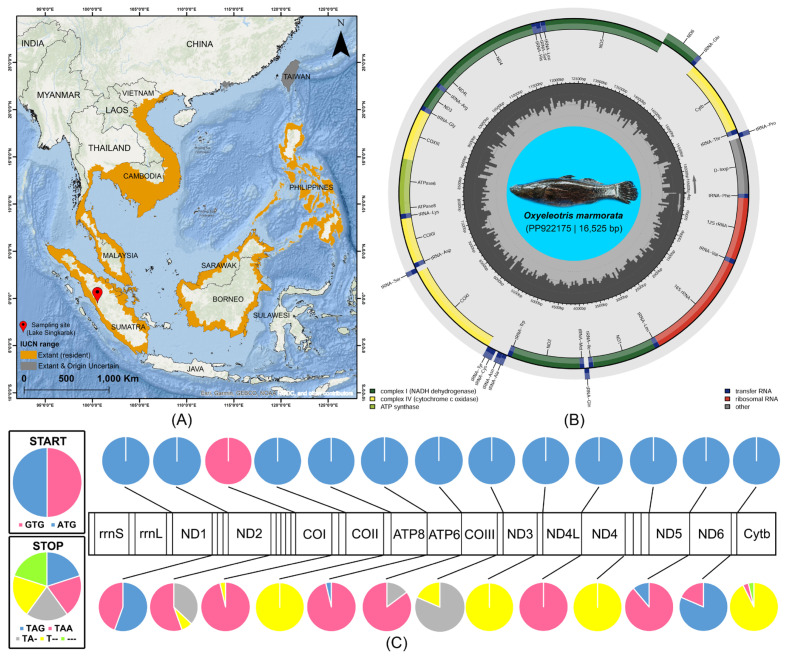
(**A**) The biogeographic distribution pattern of *O. marmorata* across Southeast and East Asia is illustrated based on the presence of extant (resident) range as well as uncertain origins. The sampling location of *O. marmorata* is marked with a red pin. (**B**) The complete mitogenome of *O. marmorata* (PP922175), obtained from its natural habitat in Lake Singkarak, Sumatra, Indonesia. The colored arcs indicate the positions of various genes, including PCGs, tRNAs, rRNAs, and CR. The species photograph was taken by Hamdani from Jakarta Technical University of Fisheries, Indonesia. (**C**) The start and stop codon usage frequencies in the 13 PCGs of *O. marmorata* and selected other Butidae and Eleotridae species are presented to illustrate genetic variation.

In this study, the complete circular mitogenome of *O. marmorata* from its native range in Indonesia, with a length of 16,525 bp, was successfully sequenced and deposited in GenBank under accession number PP922175 (Figure 1B). The comparative analysis indicated that mitogenome sizes among *Oxyeleotris* species range from 16,519 bp in *O. lineolata* (KP684140) to 16,555 bp in *O. marmorata* (KJ595342). The newly assembled genome contained 37 genes (13 PCGs, two rRNAs, 22 tRNAs, and one CR), with conserved gene content and overall gene order across all analyzed *Oxyeleotris* mitogenomes (Table 1 and Table 2). Among them, 28 genes (12 PCGs, two rRNAs, and 14 tRNAs) are encoded on the heavy (H) strand, whereas the remaining nine genes (ND6 and eight tRNAs) are located on the light (L) strand. The nucleotide composition of the newly sequenced *O. marmorata* mitogenome exhibited an A+T bias of 53.67%, comprising 28.73% A, 24.94% T, 15.47% G, and 30.86% C. In comparison, other *Oxyeleotris* mitogenomes showed slight variation, with A+T content ranging from 53.54% in *O. marmorata* (KJ595342) to 53.90% in *O. lineolata* (KP684140). The AT-skew and GC-skew of the newly sequenced mitogenome were 0.070 and −0.332, respectively. Among other *Oxyeleotris* mitogenomes, AT-skew values ranged from 0.066 in *O. marmorata* (KJ595342) and *O. lineolata* (KP684140) to 0.075 in *O. lineolata* (KP663727), while GC-skew values varied between −0.334 in *O. lineolata* (KP663727) and −0.326 in *O. lineolata* (KP684140) (Table 2).

### 2.2. Intergenic Spacer and Overlapping Regions

Analysis of intergenic spacers and gene overlaps among *Oxyeleotris* species, including three *O. marmorata* and two *O. lineolata* sequences, revealed notable variation in both the length and distribution of these features (Supplementary Table S2). The newly sequenced *O. marmorata* mitogenome (PP922175) contained 11 intergenic spacers totaling 65 bp and seven overlapping regions totaling 25 bp. Conversely, the longest intergenic spacer (37 bp) was located between trnN and trnC on the light strand, whereas the longest overlap (10 bp) was occurred between ATP8 and ATP6 on the heavy strand. Several genes, including trnF, trnL2, trnM, COII, trnG, trnR, ND4, trnH, trnL1, ND6, Cytb, and CR consistently lacked both intergenic spacers and overlaps across all analyzed mitogenomes. Conserved spacer lengths were observed between trnN and ND1 (37 bp and 3 bp, respectively) in all sequences, while other genes (trnW, trnA, trnY, trnS2, trnK, trnE) exhibited uniform intergenic spacers ranging from 1 to 5 bp. The ATP8–ATP6 gene pair displayed a conserved 10 bp overlap across all sequences. Despite these conserved patterns, interspecific and inter-accession variation was detected in several regions. Specifically, the spacer downstream of trnD measured 6 bp in *O. marmorata* (PP922175 and KJ595342), 11 bp in *O. marmorata* (KF711995), and 6 bp and 5 bp in *O. lineolata* (KP663727 and KP684140), respectively. The longest overlapping region in other mitogenomes was observed between ND5 and ND6, reaching 12 bp in *O. marmorata* (KJ595342) and *O. lineolata* (KP663727). Additional overlaps were also identified among 12S rRNA, trnV, trnI, trnQ, ND2, trnC, ATP6, COIII, ND3, ND4L, ND5, and trnT, with overlap lengths ranging from 1 to 12 bp (Supplementary Table S2).

### 2.3. Protein-Coding Genes Features

The newly sequenced of *O. marmorata* in this study contained 13 PCGs with a total length of 11,431 bp, representing 69.17% of the entire mitogenome. Among these PCGs, ATP8 was the shortest gene (168 bp), whereas ND5 was the longest (1824 bp) (Table 1). In comparison, the total lengths of PCGs in other *Oxyeleotris* mitogenomes ranged from 11,419 bp in *O. lineolata* (KP684140) to 11,453 bp in *O. marmorata* (KJ595342). The PCGs of the newly sequenced *O. marmorata* exhibited an A+T content of 53.08%, while in other species, this value ranged from 52.90% in *O. marmorata* (KJ595342) to 53.45% in *O. lineolata* (KP684140). The AT-skew and GC-skew of the PCGs in the newly sequenced mitogenome were −0.022 and −0.362, respectively. For other *Oxyeleotris* mitogenomes, AT-skew values ranged from −0.028 in *O. marmorata* (KJ595342) to −0.017 in *O. lineolata* (KP663727), and GC-skew values ranged from −0.362 in other *O. marmorata* sequences and *O. lineolata* (KP663727) to −0.358 in *O. lineolata* (KP684140) (Table 2). Moreover, the analysis of start and stop codon usage across 13 PCGs of 25 butid and eleotrid species in 27 sequences revealed consistent patterns (Supplementary Table S3). The ATG codon was the predominant initiation codon for all PCGs, except for COI, which consistently used GTG across all species examined. In contrast, stop codon usage was more variable, with TAA being the most frequently observed, appearing in ND1 (44.44%), ND2 (55.56%), COI and ATP8 (96.30%), ATP6 (85.19%), ND4L (100%), ND5 (88.89%), ND6 (18.52%), and Cytb (3.70%). The TAG codon was also detected in ND1 (55.56%), ATP8 (3.70%), ND5 (11.11%), and ND6 (81.48%). Incomplete stop codons (TA- and T--) were commonly observed in ND2, COI, COII, ATP6, COIII, ND3, ND4, and Cytb across several species. Notably, in *Gobiomorus dormitor*, the Cytb gene lacked a recognizable stop codon (---), likely due to the shorter mitogenome length in this species, resulting in the absence of the expected stop codon at the anticipated position (Figure 1C; Supplementary Table S3).

### 2.4. Substitutions Pattern and Relative Synonymous Codon Usage

**Figure 2:**
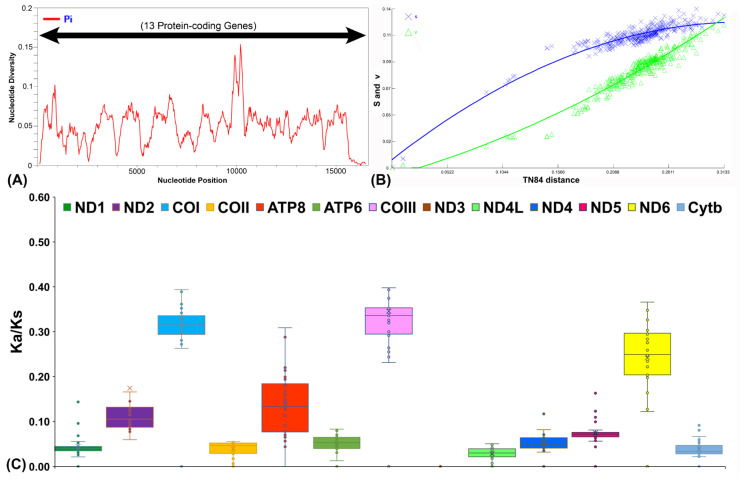
(**A**) The plot displays nucleotide diversity (π) for mitochondrial PCGs across *Oxyeleotris* species. (**B**) The line scatter plot illustrates the relationship between transitions (s) and transversions (v) with genetic divergence in PCGs, based on TN84 distances. (**C**) The box plot shows the pairwise Ka/Ks ratios for each PCG among representative Butidae and Eleotridae species, including *O. marmorata*.

**Figure 3:**
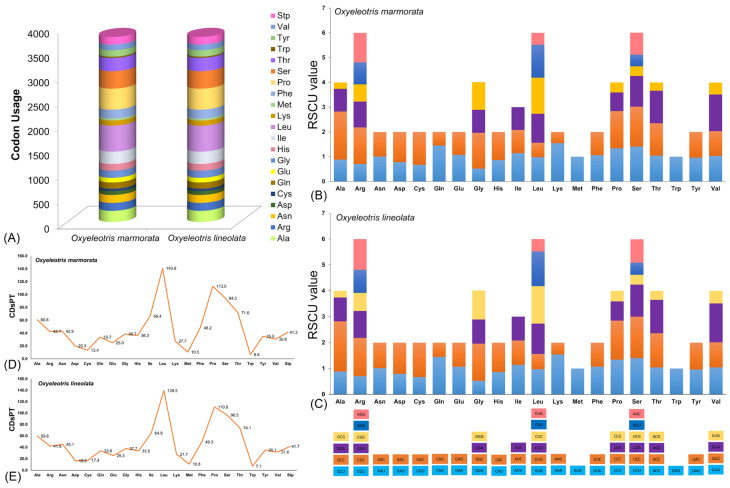
(**A**) The codon usage abundance in the mitogenomes of *Oxyeleotris* species (*O. marmorata* and *O. lineolata*). (**B**,**C**) The comparative analysis of RSCU in *Oxyeleotris* species (*O. marmorata* and *O. lineolata*), with collective RSCU values plotted on the y-axis against codons corresponding to each amino acid on the x-axis. (**D**,**E**) Codon distribution per thousand codons for all amino acids in the mitogenomes of *Oxyeleotris* species (*O. marmorata* and *O. lineolata*).

In this study, the nucleotide diversity of the assembled PCGs was assessed using a sliding window approach, revealing an average nucleotide diversity (π) of 0.04934 and identifying 1970 polymorphic sites across *Oxyeleotris* species (Figure 2A). The saturation analysis indicated no evidence of saturation for either transitions or transversions, as TN84 divergence values continued to rise across all PCGs of the *Oxyeleotris* species (Figure 2B). To investigate evolutionary pressures on homologous genes, the Ka/Ks ratio was calculated for the newly sequenced *O. marmorata* and compared with other species in the Butidae and Eleotridae families. The Ka/Ks values ranged from 0.05256 ± 0.02098 for ND4L to 1.84269 ± 0.88966 for ND3, following the order: ND4L < COII < Cytb < ND1 < ATP6 < ND4 < ND5 < ATP8 < ND2 < ND6 < COI < COIII < ND3 (Figure 2C; Supplementary Table S4). The analysis of codon usage in *O. marmorata* and *O. lineolata* revealed that arginine, leucine, and serine were the most frequently used amino acids in PCGs, whereas methionine and tryptophan occurred less frequently (Figure 3A; Supplementary Table S5). The RSCU analysis further indicated that the GCG codon for alanine was notably underrepresented compared to other codons in both species (Figure 3B,C; Supplementary Table S5). The CDsPT in *O. marmorata* and *O. lineolata* showed broadly similar patterns, although some variation was observed among species. The leucine displayed the highest CDsPT values, spanning 139.5 in *O. lineolata* to 140.9 in *O. marmorata*, whereas tryptophan and methionine were less abundant, with tryptophan varying between 6.6 (*O. marmorata*) and 7.1 (*O. lineolata*) and methionine covering 10.5 (*O. marmorata*) to 10.8 (*O. lineolata*) (Figure 3D,E; Supplementary Table S6).

### 2.5. Ribosomal and Transfer RNA Genes

The newly sequenced of *O. marmorata* (PP922175) contained two rRNA genes with a combined length of 2639 bp and 22 tRNA genes totaling 1559 bp, representing 15.97% and 9.43% of the mitogenome, respectively (Table 1). The comparative analysis across available *Oxyeleotris* mitogenomes revealed that total rRNA length ranged from 2615 bp in *O. lineolata* (KP684140) to 2640 bp in *O. lineolata* (KP663727). The rRNA genes exhibited an A+T compositional bias, with values ranging from 53.28% in *O. marmorata* (PP922175) to 53.54% in *O. lineolata* (KP684140). The rRNA AT-skew values varied from 0.239 in *O. lineolata* (KP684140) to 0.268 in *O. lineolata* (KP663727), while GC-skew values ranged from −0.151 in *O. lineolata* (KP663727) to −0.141 in *O. marmorata* (KJ595342). In comparison, the total tRNA length among *Oxyeleotris* species ranged from 1485 bp in *O. marmorata* (KF711995) to 1575 bp in *O. marmorata* (KJ595342). The tRNA genes also demonstrated A+T bias, with proportions ranging from 54.95% in *O. marmorata* (KF711995) to 55.16% in *O. marmorata* (PP922175) and *O. lineolata* (KP663727). The AT-skew values for tRNAs ranged from 0.025 in *O. lineolata* (KP684140) to 0.156 in *O. marmorata* (PP922175), while the GC-skew values ranged from −0.147 in *O. marmorata* (PP922175) to 0.037 in *O. lineolata* (KP684140) (Table 2). Furthermore, almost all *Oxyeleotris* species exhibited fully conserved anticodon sequences across the complete set of 22 tRNAs, except for trnW in *O. marmorata* (KJ595342), which lacked a detectable anticodon (Supplementary Table S7). The majority of tRNAs in the newly sequenced *O. marmorata* (PP922175) conformed to the canonical cloverleaf secondary structure, with the exception of trnS1, which lacked a functional dihydrouridine (DHU) arm. The structural assessment revealed that 12 tRNAs (trnF, trnQ, trnW, trnA, trnN, trnC, trnY, trnS2, trnD, trnG, trnE, and trnP) contained both canonical Watson–Crick base pairing (A–T and G≡C) and wobble pairing (G–T), whereas the remaining 10 tRNAs (trnV, trnL2, trnI, trnM, trnK, trnR, trnH, trnS1, trnL1, and trnT) exhibited only canonical Watson–Crick pairing (Supplementary Figure S1).

### 2.6. Characteristics of Control Region

**Figure 4:**
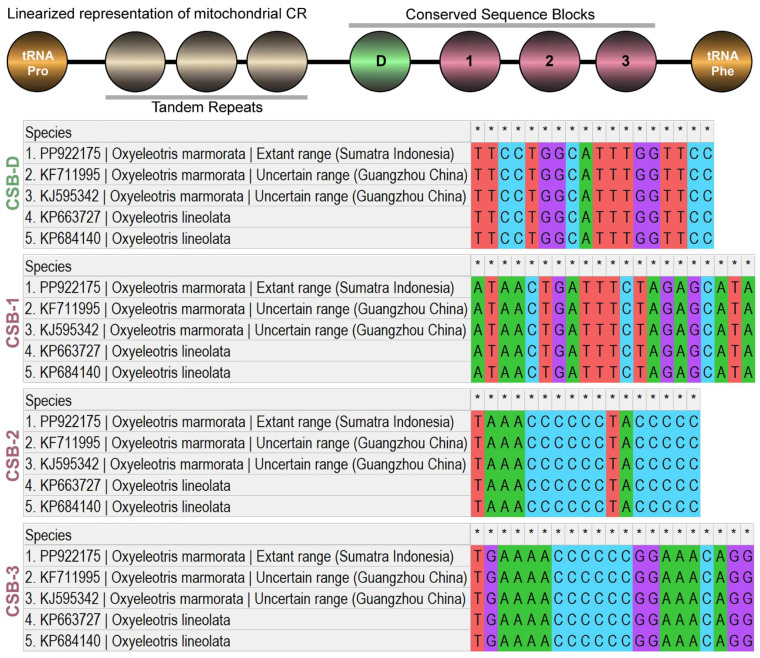
The schematic evaluation of nucleotide composition and the length of conserved domains within the CR of *O. marmorata* from Sumatra (Indonesia), two *O. marmorata* sequences from Guangzhou (China), and two *O. lineolata* sequences. A general linear representation is shown at the top, with highly conserved nucleotides indicated by small black star symbols.

The CR in the newly sequenced of *O. marmorata* was 856 bp in length, accounting for 5.18% of the total mitogenome (Table 1). The comparative analysis across different *Oxyeleotris* mitogenomes revealed slight variation in CR length, ranging from 856 bp in *O. marmorata* (KF711995) and *O. lineolata* (KP663727) to 858 bp in *O. lineolata* (KP684140). The CR of the newly sequenced *O. marmorata* also exhibited an A+T bias of 60.98%, with an AT-skew of −0.011 and a GC-skew of −0.144. By comparison, other *Oxyeleotris* mitogenomes showed A+T bias values in the CR ranging from 59.09% in *O. lineolata* (KP684140) to 61.21% in *O. marmorata* (KF711995) and *O. lineolata* (KP663727). The AT-skew values ranged from −0.015 in *O. marmorata* (KF711995) and *O. lineolata* (KP663727) to −0.004 in *O. marmorata* (KJ595342), while the GC-skew values ranged from −0.146 in *O. marmorata* (KJ595342) and *O. lineolata* (KP684140) to −0.139 in *O. marmorata* (KF711995) and *O. lineolata* (KP663727) (Table 2). Additionally, detailed mitogenomic analysis of *O. marmorata* and *O. lineolata* identified four conserved sequence blocks (CSBs) within the CR: CSB-D, CSB-1, CSB-2, and CSB-3. Although these elements are conserved, the comparative analysis revealed notable nucleotide variability, particularly in CSB-1 and CSB-3 (21 bp each), whereas CSB-D and CSB-2 were shorter, measuring 18 bp and 17 bp, respectively (Figure 4). Furthermore, no tandem repeats were detected in the CR among *Oxyeleotris* congeners.

### 2.7. Haplotype Diversity and Genetic Divergence

**Figure 5:**
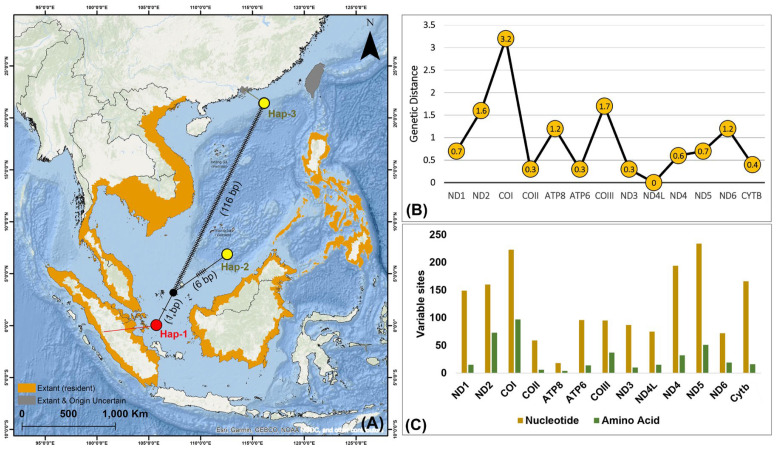
(**A**) This map presents the geographical distribution of *O. marmorata* across Southeast and East Asia. The TCS haplotype network illustrates the genetic relationships among all *O. marmorata* haplotypes derived from the native habitat in Lake Singkarak, Sumatra, Indonesia (indicated by red circles), and the introduced habitat in Guangzhou, China (indicated by yellow circles). The circle sizes reflect the frequency of each haplotype, while the number of mutations between haplotypes is shown in parentheses. The median vectors representing hypothetical haplotypes are depicted as black circles. (**B**) The line graph displays the frequency of K2P genetic distances across 13 PCGs, estimated from the mitogenomes of *O. marmorata*, including one individual from the native habitat and two sequences from the introduced habitat. (**C**) The bar chart illustrates the variation in the number of nucleotide and amino acid sites across the 13 PCGs, compared between two *Oxyeleotris* species.

The haplotype analysis of *O. marmorata* from its native habitat (Lake Singkarak, West Sumatra, Indonesia) and introduced habitat (Guangzhou, China) identified three haplotypes, with 123 segregating sites, a haplotype diversity (Hd) of 1.0000, and nucleotide diversity (π) of 0.6667 (Figure 5A; Supplementary Table S8). The genetic distance analyses of the 13 PCGs between *O. marmorata* from native and introduced habitats revealed the highest pairwise divergence in COI (3.2%) and the lowest in ND4L (0%) (Figure 5B; Supplementary Table S9). However, the comparative analysis of variable sites across the 13 PCGs between *O. marmorata* and *O. lineolata* demonstrated gene-specific patterns of nucleotide and amino acid variation. Notably, ND5 had the highest number of variable nucleotide sites (234), followed by COI (223) and ND4 (194), while at the amino acid level, COI exhibited the greatest variation (97), followed by ND2 (73) and ND5 (51). In contrast, ATP8 displayed the fewest variations, with only 18 nucleotide and 4 amino acid variable sites (Figure 5C; Supplementary Table S9).

### 2.8. Matrilineal Phylogenetic Relationships

**Figure 6:**
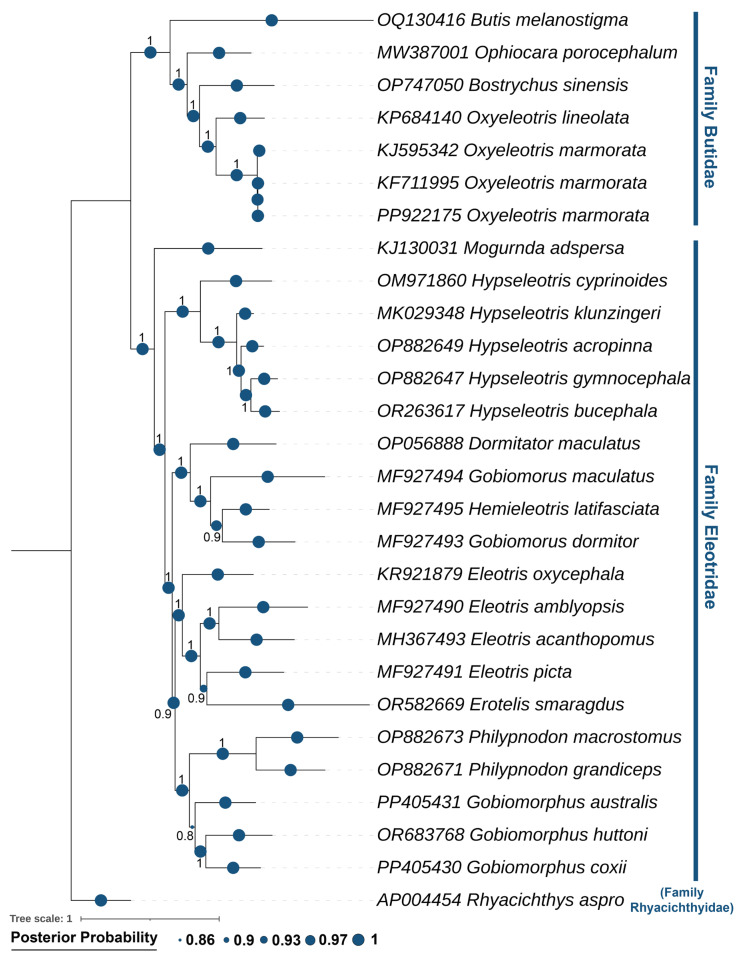
The Bayesian phylogenetic tree, inferred from 13 concatenated PCGs, distinctly places *O. marmorata* within the Butidae clade, separating it from other Butidae and Eleotridae species. The posterior probability support values are indicated by blue circles at each node.

The mitogenome-based phylogenetic analysis was performed using 13 PCGs from the newly sequenced of *O. marmorata* along with other related species from the Butidae and Eleotridae families. The phylogenetic trees constructed using BA and ML methods resolved the species into distinct clades with strong statistical support. The analysis demonstrated that *O. marmorata* shares a close evolutionary relationship with *O. lineolata*, a tropical congener distributed across northern Australia and New Guinea, with both species forming a monophyletic group. This clade was sister to *B. sinensis*, which has a broad Indo-West Pacific distribution from eastern India to the Philippines, Vietnam, and China, extending north to southern Japan and south to northern Australia. Beyond the *Oxyeleotris* and *Bostrychus* genera, the phylogeny also supported matrilineal relationships within other Butidae genera, including *Butis* and *Ophiocara*, and within Eleotridae, covering *Mogurnda*, *Hypseleotris*, *Dormitator*, *Gobiomorus*, *Hemieleotris*, *Eleotris*, *Erotelis*, *Philypnodon*, and *Gobiomorphus*. Notably, the cladistic analysis also uncovered a non-monophyletic structure within the genus *Gobiomorus*, with *G. maculatus* and *G. dormitor* forming separate lineages, suggesting that further investigation is required (Figure 6; Supplementary Figure S2).

## 3. Discussion

### 3.1. Mitogenomic Profiling

The newly characterized mitogenome of *O. marmorata* comprises 37 genes, with 28 on the heavy strand and nine on the light strand. The gene order and strand distribution are conserved relative to other teleosts, and the pronounced A+T bias aligns with the hydrophobic properties of mitochondrial proteins [24,28,32,33]. Comparative analyses of *Oxyeleotris* species revealed the longest intergenic spacer and overlapping regions between trnN–trnC and ATP8–ATP6, respectively, a pattern characteristic of canonical mitochondrial genomes in teleost fishes [34]. The conserved overall mitogenome architecture suggests that nucleotide composition and gene organization can serve as reliable markers for phylogenetic inference within *Oxyeleotris* [25,29]. The PCGs also follow a conserved pattern, with ATP8 as the shortest and ND5 as the longest, consistent with prior observation in another goby [35]. Most PCGs in Butidae and the closely related Eleotridae start with the canonical ATG codon, whereas COI uses GTG, reflecting conserved selective pressures on mitochondrial translation [27,36]. Conversely, the stop codon usage varies, with six PCGs ending with complete codons (TAA/TAG) and the remainder exhibiting truncated codons (TA-/T--), presumably completed via polyadenylation during RNA processing [37,38]. These patterns underscore a conserved initiation mechanism alongside flexible termination strategies, which may facilitate mitogenomic evolution and adaptation.

Furthermore, the Ka/Ks analysis in Butidae and its closest related family Eleotridae showed that all PCGs have ratios <1, indicating strong purifying selection against deleterious nonsynonymous mutations and no evidence of positive selection, reflecting evolutionary stability in mitochondrial proteins [39,40]. The RSCU analysis indicates differential codon preferences, which may reflect translational optimization in *O. marmorata* and provide a framework for studying gene expression dynamics and adaptive evolution within the Butidae [41]. The rRNA genes (12S and 16S) of *O. marmorata* exhibit the typical vertebrate organization, separated by trnV on the heavy strand [36]. Conversely, most tRNA genes of this species display canonical cloverleaf structures, except trnS1, which lacks the DHU arm, a typical mitochondrial feature that preserves function and reflects evolutionary adaptation [38]. The structural integrity of the WANCY tRNAs in *O. marmorata* likely supports efficient mitochondrial translation and gene expression [42,43]. In addition, the CR among the *Oxyeleotris* species harbors conserved sequence blocks (CSB-D, CSB-1, CSB-2, CSB-3), consistent with their regulatory role in mitochondrial DNA replication termination [25,27]. The observed variability in CR sequences, together with potential structural mechanisms such as dimer formation and differential strand replication, may underlie mitogenomic structural diversity and contribute to the evolutionary dynamics of *O. marmorata* and related species [29,33]. Overall, the findings reveal structural conservation alongside functional variability in the *O. marmorata* mitogenome, providing a framework for understanding evolutionary constraints, translational dynamics, and mitochondrial adaptation in Gobiiformes.

### 3.2. Genetic Diversity and Matrilineal Phylogenetic Insights

The haplotype analysis in the present study indicated clear genetic differences between the native *O. marmorata* from Lake Singkarak, West Sumatra, Indonesia, and the introduced localities in Guangzhou, China. The Indonesian haplotype differed from the two Chinese haplotypes, exhibiting nucleotide variations ranging from 7 to 117 base pairs. These differences likely reflect multiple-source introductions from diverse geographic regions or the accumulation of mutations under aquaculture conditions [44]. Moreover, artificial selection, novel environmental pressures, and genetic isolation within captive systems may further accelerate haplotype differentiation [45,46]. These findings suggest that species translocations can generate new patterns of genetic variation through the combined effects of ecological and anthropogenic factors [23]. Furthermore, the analysis of the 13 PCGs revealed considerable variation in genetic divergence among loci. The COI gene exhibited the highest levels of nucleotide and amino acid variation, confirming its suitability as a molecular marker for distinguishing species, resolving taxonomic relationships, and supporting further phylogeographic studies of *O. marmorata* in Southeast and East Asia [47]. In contrast, highly conserved genes in *O. marmorata* such as ND4L and ATP8 displayed minimal variation, likely constrained by essential mitochondrial functions, making them suitable for deep evolutionary or comparative analyses [48,49]. Additionally, by integrating multiple genes and non-coding regions, the mitogenome-based phylogeny in this study provides a comprehensive view of matrilineal relationships within *Oxyeleotris* and closely related goby species in Butidae and Eleotridae [5,6,28]. The two *Oxyeleotris* species, *O. lineolatus* and *O. marmorata*, formed a monophyletic clade with close genetic affinity, whereas *B. sinensis* diverged earlier, in agreement with previous phylogenetic studies [6,21,22,35]. These results highlight the utility of mitogenomic data for elucidating potential ancestral lineages and resolving morphological inconsistencies reported among ichthyologists [29,38]. Considering the diversity and broad distribution of the remaining 17 *Oxyeleotris* species, additional mitogenomic studies are warranted to further investigate their speciation processes and adaptive evolution across heterogeneous aquatic habitats.

### 3.3. Study Limitations, Conservation Implications, and Future Perspectives

The present study reports a single complete mitochondrial genome generated from one individual representing the native Indonesian range of *O. marmorata*, providing an initial matrilineal genomic baseline for comparative analyses. However, reliance on a single specimen may not capture the full extent of mitogenomic diversity within Indonesia or across Southeast Asia; accordingly, the representativeness and broader applicability of the findings should be interpreted with caution. Despite this limitation, the dataset constitutes a valuable reference for characterizing mitogenomic features and inferring phylogenetic relationships. In addition, the introduction of non-native species, including *O. marmorata*, poses a significant threat to biodiversity by competing with and potentially displacing native gobies [19,50]. Such introductions can also lead to hybridization between native and introduced populations, which may compromise genetic integrity, reduce adaptive uniqueness, and alter ecological distributions and population dynamics [51,52]. Hence, we recommend that future studies incorporate larger sample sizes and generate complementary molecular datasets, including complete mitogenomes, nuclear markers, microsatellites, and single-nucleotide polymorphisms, to resolve fine-scale genetic variation, detect hybridization and more robustly infer population structure. Collectively, such integrative genomic approaches can strengthen conservation strategies by providing robust inferences of genetic diversity and demographic structure across both native and non-native ecosystems.

## 4. Materials and Methods

### 4.1. Sample Collection, Identification, and Preservation

A single wild specimen of *O. marmorata* was harvested from Lake Singkarak, West Sumatra, Indonesia (coordinates 0°33′52″ S, 100°32′11″ E), using a 5/8-inch gill net (Figure 1A). The identification was conducted following taxonomic keys from the previous investigations [14,53]. The specimen exhibited seven dorsal spines, nine dorsal soft rays, one anal spine, eight anal soft rays, and 60–65 predorsal scales, with no ocellus on the caudal peduncle. The specimen was euthanized by administering 2-phenoxyethanol at a final concentration of 600 µL L^−1^ directly into the aquarium, and after complete cessation of movement, it was rinsed three times with Milli-Q water [54]. Further, approximately 20 g of apical muscle tissue was aseptically collected and preserved in 95% molecular-grade ethanol in a 2 mL centrifuge tube, which was sealed with parafilm to prevent DNA degradation and microbial contamination and stored at −20 °C. The samples were subsequently transferred to the Molecular Physiology Laboratory at Pukyong National University, Busan, Republic of Korea, for molecular analyses. For archival purposes, the specimen was cataloged and fixed in 10% formaldehyde under voucher number ‘IDN5’ at the Jakarta Technical University of Fisheries (Pariaman Campus), Ministry of Marine Affairs and Fisheries, Indonesia. The distribution records of *O. marmorata*, including confirmed resident and those of uncertain origin, were mapped using IUCN shapefiles and visualized with ArcGIS version 10.6 to illustrate biogeographical patterns across Southeast and East Asia (Figure 1A). The species under this study is locally utilized as a food fish and is currently classified as “Least Concern” on the IUCN Red List of Threatened Species (https://www.iucnredlist.org/, accessed on 24 November 2025). Nevertheless, all experiments were conducted in accordance with institutional guidelines, approved by the Pukyong National University Institutional Animal Care and Use Committee (IACUC) under code PKNUIACUC-2025-16 dated 18 February 2025, and adhered to the ARRIVE 2.0 guidelines (https://arriveguidelines.org/, accessed on 24 November 2025) [55].

### 4.2. Genomic DNA Extraction and COI Amplification

The genomic DNA was extracted using the AccuPrep^®^ Genomic DNA Extraction Kit (Bioneer, Daejeon, Republic of Korea), following the standard procedure outlined by the manufacturer. Approximately 30 mg of tissue was homogenized in 600 μL of 1× lysis buffer using a Tissue Lyser II (Qiagen, Hilden, Germany) for 60 s. Cell lysis and protein degradation were achieved by adding 100 μL of sodium dodecyl sulfate (SDS) and 20 μL of proteinase K, followed by incubation at 60 °C for 12 h. The DNA precipitation was facilitated by adding 500 μL of GC buffer and 300 μL of isopropanol. The mixture was then transferred to a column tube and centrifuged at 8000 rpm for 1 min. The contaminants were removed through sequential washes with buffer 1 and buffer 2, and purified DNA was eluted in 50 μL of TE buffer. The DNA concentration and purity were assessed using a NanoDrop spectrophotometer (Thermo Fisher Scientific, D1000, Waltham, MA, USA). For species verification, a partial fragment of the mitochondrial COI gene was amplified by polymerase chain reaction (PCR) using the universal primers Fish-BCH (5′-TCAACYAATCAYAAAGATATYGGCAC-3′) as the forward primer and Fish-BCL (5′-ACTTCYGGGTGRCCRAARAATCA-3′) as the reverse primer [56]. The PCR was executed in a total volume of 30 µL and contained 1 µL of each primer (10 pmol), 0.9 μL of 3% dimethyl sulfoxide (DMSO), 19.9 μL of sterilized deionized water, 3 μL of 10× ExTaq Buffer, 0.2 μL of Ex Taq HS, 3 μL of dNTPs, and 1 μL of a 1:10 diluted DNA template. The thermal cycling was performed on a Takara thermal cycler, beginning with initial denaturation at 94 °C for 3 min, followed by 40 cycles of 94 °C for 30 s, 50 °C for 30 s, 72 °C for 1 min, and a final extension at 72 °C for 5 min. The PCR products were purified using the AccuPrep^®^ PCR/Gel Purification Kit (Bioneer, Daejeon, Republic of Korea) and sequenced bidirectionally on a 96-capillary ABI PRISM 3730XL Analyzer at Macrogen in Daejeon, Republic of Korea (https://dna.macrogen.com/, accessed on 24 November 2025). The sequencing chromatograms were filtered for noise using SeqScanner v1.0 (Applied Biosystems, Foster City, CA, USA), and COI sequences were verified via nucleotide BLAST searches against the GenBank database (https://blast.ncbi.nlm.nih.gov, accessed on 24 November 2025).

### 4.3. Mitogenome Sequencing and Assembly

Next-generation sequencing (NGS) was performed using paired-end reads (2 × 150 bp) on the NovaSeq platform at Macrogen (Daejeon, Republic of Korea) to generate the complete mitogenome of *O. marmorata*. The library preparation followed the TruSeq Nano DNA High-Throughput Library Prep Kit protocol (Illumina, Inc., San Diego, CA, USA). Briefly, 100 ng of genomic DNA was fragmented via adaptive focused acoustics (Covaris, Woburn, MA, USA) to produce double-stranded DNA with blunt ends and 5′ phosphorylation. Fragmented DNA underwent end repair, and then ligated with TruSeq DNA UD Indexing adapters, which were modified with a single 3′ adenine overhang, and subsequent size selection using a bead-based method. The library was then purified, enriched by PCR, and quantified via qPCR according to the KAPA Library Quantification Kit guidelines for Illumina sequencing platforms. The library quality was assessed using the Agilent 4200 TapeStation with D1000 ScreenTape (Agilent Technologies, Santa Clara, CA, USA). Furthermore, high-quality sequencing reads were used to assemble the mitogenome in Geneious Prime v2023.0.1 [57], with alignment guided by the previously published *O. marmorata* mitogenome from China (GenBank accession no. KJ595342). For precise sequence assembly, the overlapping regions were refined and verified using MEGA v12 [58]. Gene positions, boundaries, and orientations were independently confirmed using two annotation platforms: MITOS2 (integrated with the Galaxy web server, https://usegalaxy.eu, accessed on 24 November 2025) and MitoAnnotator (http://mitofish.aori.u-tokyo.ac.jp/annotation/input/, accessed on 24 November 2025) [26,59]. The PCGs were further validated by examining their predicted amino acid sequences using the Open Reading Frame Finder tool (https://www.ncbi.nlm.nih.gov/orffinder/, accessed on 24 November 2025) under the standard vertebrate mitochondrial genetic code. The final annotated mitogenome of *O. marmorata*, including strand orientation and gene features, was submitted to GenBank via the Sequin platform.

### 4.4. Mitogenome Characterization, Comparative Analyses, and Genetic Diversity

In this investigation, the mitogenome of *O. marmorata* from its native range in Indonesia was visualized using MitoAnnotator. The comparative analyses were performed by aligning the newly generated sequence with available *Oxyeleotris* mitogenomes from GenBank (Supplementary Table S1), including two previously published *O. marmorata* sequences from China (KF711995, KJ595342) and two *O. lineolata* sequences (KP663727, KP684140) [19,20,22,60]. Intergenic spacers and overlapping regions among *Oxyeleotris* species were manually quantified in Microsoft Excel v2016. The nucleotide composition of PCGs, rRNAs, tRNAs, and CR was analyzed in MEGA v12, while the A-T and G-C composition were calculated using AT-skew = (A − T)/(A + T) and GC-skew = (G − C)/(G + C) [61]. The start and stop codons of PCGs across 27 sequences representing 25 Butidae species and the closely related Eleotridae were annotated using the Open Reading Frame Finder tool (Supplementary Table S1). The nucleotide diversity (π) among *Oxyeleotris* species was assessed using a sliding window analysis in DnaSP v6.0 with a window size of 200 bp and a step size of 25 bp [62]. The transition–transversion saturation across PCG codon positions was evaluated in DAMBE v6 [63]. The rates of nonsynonymous (Ka) and synonymous (Ks) substitutions were also calculated in DnaSP v6.0. The codon usage patterns, including amino acid frequency, Relative Synonymous Codon Usage (RSCU), and Codon Distribution per Thousand Codons (CDsPT), were similarly examined. The tRNA secondary structures of *O. marmorata* were predicted using Aragorn on the Galaxy platform [64]. The conserved CR motifs among *Oxyeleotris* species were identified through CLUSTAL X alignments and verified against previously characterized conserved domains [27,65]. The Tandem Repeats Finder (https://tandem.bu.edu/trf/trf.html, accessed on 24 November 2025) was also used to detect repetitive elements due to their relevance in population level investigation [66]. Further, the genetic diversity indices of *O. marmorata*, including the number of haplotypes, haplotype diversity (Hd), and nucleotide diversity (π), were estimated from 13 PCGs using DnaSP v6.0. The haplotype relationships were visualized using a TCS network constructed in POPART v.1.7 [67,68]. The genetic distances between *O. marmorata* from native and introduced habitats were calculated using the Kimura 2-parameter (K2P) model in MEGA v12, based on concatenated sequences of the 13 PCGs. Lastly, variable nucleotide and amino acid sites between *O. marmorata* and *O. lineolata* were also examined to identify species-specific molecular signatures and quantify genetic differentiation.

### 4.5. Dataset Configuration and Phylogenetic Framework

The phylogenetic dataset comprised 27 mitogenome representing 25 valid species from the Butidae and Eleotridae families, including the newly assembled *O. marmorata* mitogenome from its native distribution in Indonesia and 26 sequences retrieved from GenBank (Supplementary Table S1). The mitogenome of *Rhyacichthys aspro* (GenBank accession no. AP004454) was selected as the outgroup. The phylogenetic construction was performed using Bayesian inference (BA) and Maximum Likelihood (ML) approaches based on a concatenated alignment of the 13 PCGs generated in iTaxoTools v0.1 [69]. The best-fit evolutionary model, GTR+G+I, was identified using PartitionFinder v2 under the Bayesian Information Criterion [70]. The BA tree was implemented in MrBayes v3.1.2 using the Metropolis-coupled Markov Chain Monte Carlo (MCMC) framework with nst = 6. The analysis was run for 10,000,000 generations with sampling every 100 generations, and the first 25% of sampled trees were discarded as burn-in. The convergence was confirmed when the average standard deviation of split frequencies reached ≤0.01 and the Potential Scale Reduction Factor (PSRF) values approached 1 across all estimated parameters [71]. Conversely, the ML tree was generated in PhyML v3.0 with 1000 bootstrap replicates [72]. The final trees visualization and annotation were performed using iTOL v7 [73].

## 5. Conclusions

This study provides an in-depth analysis of the complete mitogenome of *O. marmorata* from its native range in Indonesia, elucidating its gene organization and structural architecture. The phylogenetic inference further offers insights into the evolutionary dynamics of *Oxyeleotris* species and their taxonomic placement within the Butidae and its closest related family, Eleotridae. The structural variations in the mitogenome are shown to contribute to evolutionary divergence, informing species differentiation and adaptation. The findings supply critical genetic data applicable to systematics, molecular taxonomy, conservation genetics, and the sustainable management of goby populations. This work underscores the need to establish a comprehensive mitogenomic database for the remaining 17 *Oxyeleotris* species. Future research should incorporate broader sampling of *O. marmorata* from both wild and cultured populations across diverse regions of Southeast and East Asia to increase sample size and improve population representativeness. In addition, the use of complementary nuclear markers, such as microsatellites and single-nucleotide polymorphisms, will be essential to resolve fine-scale genetic variation, detect hybridization, and more robustly infer population structure and local adaptation.

## Figures and Tables

**Table 1:** Annotated genes in the newly sequenced mitochondrial genome of *O. marmorata* (PP922175), including gene positions, strand orientation, lengths, and intergenic nucleotide regions.

Genes	Start	End	Strand	Size (bp)	Intergenic Nucleotide	Anti-Codon	Start Codon	Stop Codon
tRNA-Phe (F)	1	68	H	68	0	GAA		
12S rRNA	69	1022	H	954	0			
tRNA-Val (V)	1023	1094	H	72	−1	TAC		
16S rRNA	1094	2778	H	1685	1			
tRNA-Leu (L2)	2780	2855	H	76	0	TAA		
ND1	2856	3830	H	975	3		ATG	TAG
tRNA-Ile (I)	3834	3903	H	70	−1	GAT		
tRNA-Gln (Q)	3903	3973	L	71	−1	TTG		
tRNA-Met (M)	3973	4041	H	69	0	CAT		
ND2	4042	5087	H	1046	0		ATG	TA-
tRNA-Trp (W)	5088	5159	H	72	3	TCA		
tRNA-Ala (A)	5163	5231	L	69	1	TGC		
tRNA-Asn (N)	5233	5305	L	73	37	GTT		
tRNA-Cys (C)	5343	5408	L	66	0	GCA		
tRNA-Tyr (Y)	5409	5479	L	71	1	GTA		
COI	5481	7034	H	1554	0		GTG	TAA
tRNA-Ser (S2)	7035	7105	L	71	3	TGA		
tRNA-Asp (D)	7109	7180	H	72	6	GTC		
COII	7187	7877	H	691	0		ATG	T--
tRNA-Lys (K)	7878	7953	H	76	1	TTT		
ATP8	7955	8122	H	168	−10		ATG	TAA
ATP6	8113	8795	H	683	0		ATG	TA-
COIII	8796	9580	H	785	0		ATG	TA-
tRNA-Gly (G)	9581	9652	H	72	0	TCC		
ND3	9653	10,001	H	349	0		ATG	T--
tRNA-Arg (R)	10,002	10,070	H	69	0	TCG		
ND4L	10,071	10,367	H	297	−7		ATG	TAA
ND4	10,361	11,741	H	1381	0		ATG	T--
tRNA-His (H)	11,742	11,810	H	69	0	GTG		
tRNA-Ser (S1)	11,811	11,879	H	69	4	GCT		
tRNA-Leu (L1)	11,884	11,956	H	73	0	TAG		
ND5	11,957	13,795	H	1824	−4		ATG	TAA
ND6	13,792	14,313	L	522	0		ATG	TAG
tRNA-Glu (E)	14,314	14,382	L	69	5	TTC		
Cytb	14,388	15,528	H	1141	0		ATG	T--
tRNA-Thr (T)	15,529	15,600	H	72	−1	TGT		
tRNA-Pro (P)	15,600	15,669	L	70	0	TGG		
Control region	15,670	16,525		856				

**Table 2:** Nucleotide composition of mitogenomes across different *Oxyeleotris* species.

Species Name (GenBank Accession Number)	Size (bp)	A%	T%	G%	C%	A+T%	AT-Skew	GC-Skew
Complete mitogenome								
*O. marmorata* (PP922175)	16,525	28.73	24.94	15.47	30.86	53.67	0.070	−0.332
*O. marmorata* (KF711995)	16,525	28.71	24.95	15.47	30.86	53.66	0.070	−0.332
*O. marmorata* (KJ595342)	16,555	28.54	25.00	15.54	30.92	53.54	0.066	−0.331
*O. lineolata* (KP663727)	16,522	28.92	24.89	15.38	30.81	53.81	0.075	−0.334
*O. lineolata* (KP684140)	16,519	28.74	25.16	15.47	30.42	53.90	0.066	−0.326
PCGs								
*O. marmorata* (PP922175)	11,431	25.96	27.13	14.96	31.96	53.08	−0.022	−0.362
*O. marmorata* (KF711995)	11,432	25.94	27.12	14.98	31.97	53.05	−0.022	−0.362
*O. marmorata* (KJ595342)	11,453	25.71	27.20	15.04	32.06	52.90	−0.028	−0.362
*O. lineolata* (KP663727)	11,445	26.16	27.09	14.92	31.83	53.25	−0.017	−0.362
*O. lineolata* (KP684140)	11,419	26.19	27.25	14.94	31.61	53.45	−0.020	−0.358
rRNAs								
*O. marmorata* (PP922175)	2639	33.46	19.82	20.05	26.68	53.28	0.256	−0.142
*O. marmorata* (KF711995)	2637	33.49	19.83	19.98	26.70	53.32	0.256	−0.144
*O. marmorata* (KJ595342)	2637	33.52	19.87	20.02	26.58	53.39	0.256	−0.141
*O. lineolata* (KP663727)	2640	33.86	19.55	19.77	26.82	53.41	0.268	−0.151
*O. lineolata* (KP684140)	2615	33.15	20.38	19.81	26.50	53.54	0.239	−0.145
tRNAs								
*O. marmorata* (PP922175)	1559	31.88	23.28	19.11	25.72	55.16	0.156	−0.147
*O. marmorata* (KF711995)	1485	28.62	26.33	23.10	21.95	54.95	0.042	0.025
*O. marmorata* (KJ595342)	1575	28.38	26.60	23.17	21.84	54.98	0.032	0.030
*O. lineolata* (KP663727)	1570	28.47	26.69	22.93	21.91	55.16	0.032	0.023
*O. lineolata* (KP684140)	1557	28.20	26.85	23.31	21.64	55.04	0.025	0.037
CRs								
*O. marmorata* (PP922175)	856	30.14	30.84	16.71	22.31	60.98	−0.011	−0.144
*O. marmorata* (KF711995)	856	30.14	31.07	16.71	22.08	61.21	−0.015	−0.139
*O. marmorata* (KJ595342)	857	30.34	30.57	16.69	22.40	60.91	−0.004	−0.146
*O. lineolata* (KP663727)	856	30.14	31.07	16.71	22.08	61.21	−0.015	−0.139
*O. lineolata* (KP684140)	858	29.25	29.84	15.97	21.45	59.09	−0.010	−0.146

## Data Availability

The mitogenomic sequence data supporting the findings of this study are publicly available in GenBank (NCBI) at https://www.ncbi.nlm.nih.gov/ under accession number PP922175, accessed on 24 November 2025.

## Supplementary Materials

The following supporting information can be downloaded at: https://www.mdpi.com/article/10.3390/ijms27010140/s1.
