# Supplementary material for: Mitogenomic Characterization, Genetic Diversity, and Matrilineal Phylogenetic Insights of the Marbled Goby (Oxyeleotris marmorata) from Its Native Range in Indonesia
Source: Int J Mol Sci. 2025 Dec 22;27(1):140. doi: 10.3390/ijms27010140 (PMC12785931; doi:10.3390/ijms27010140)
Supplement: Supplementary file 1 [file ijms-27-00140-s001.zip › ijms-4035871-supplementary.pdf]

Supplementary Material

Mitogenomic Characterization, Genetic Diversity, and Matrilineal Phylogenetic Insights of the Marbled Goby (*Oxyeleotris marmorata*) from its Native Range in Indonesia

Sarifah Aini[1,†], Angkasa Putra[1,†], Hye-Eun Kang[2], Mira Maulita[3], Sang Van Vu[4], Hyun-Woo Kim[5,6,7,8], Kyoungmi Kang[9*], Shantanu Kundu[1,9,10*]

[1]Interdisciplinary Program of Marine and Fisheries Sciences and Convergent Technology, Pukyong National University, Busan 48513, Republic of Korea.

[2]Institute of Marine Life Science, Pukyong National University, Busan 48513, Republic of Korea.

[3]Jakarta Technical University of Fisheries, Ministry of Marine Affairs and Fisheries, Jakarta 12520, Republic of Indonesia.

[4]Faculty of Biology, University of Science, Vietnam National University, Hanoi, Hanoi 11400, Vietnam.

[5]Department of Marine Biology, Pukyong National University, Busan 48513, Republic of Korea.

[6]Research Center for Marine Integrated Bionics Technology, Pukyong National University, Busan 48513, Republic of Korea.

[7]Marine Integrated Biomedical Technology Center, National Key Research Institutes in Universities, Pukyong National University, Busan 48513, Republic of Korea.

[8]Department of Biology, Faculty of Science and Technology, Airlangga University, Surabaya 60115, Republic of Indonesia.

[9]Ocean and Fisheries Development International Cooperation Institute, College of Fisheries Science, Pukyong National University, Busan 48513, Republic of Korea.

[10]International Graduate Program of Fisheries Science, Pukyong National University, Busan 48513, Republic of Korea.

[†]These authors contributed equally to this work.

*Corresponding author: kangkm@pknu.ac.kr (K.K.); shantanu1984@pknu.ac.kr and shantanu1984@gmail.com (S.K.)

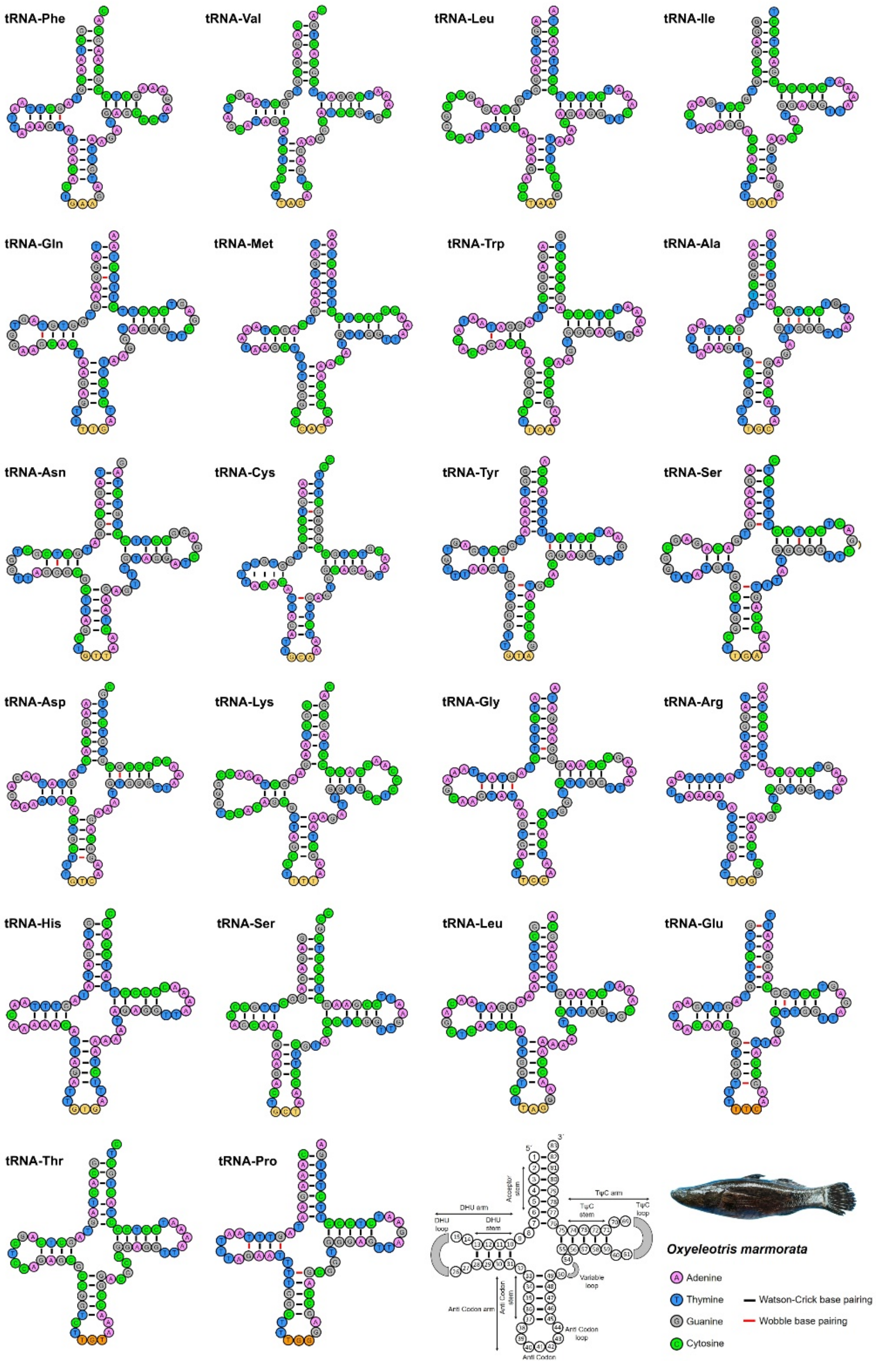

Figure S1. The secondary structures of the 22 tRNAs in the *O. marmorata* mitogenome illustrating nucleotide composition and structural variation. Each tRNA is labeled with its corresponding three-letter amino acid code according to IUPAC-IUB conventions. The final consensus structure highlights nucleotide positions and the typical stem–loop architecture of the tRNAs. Watson–Crick base pairings and wobble pairs are shown as black and red bars, respectively. The species photograph was taken by Hamdani from the Jakarta Technical University of Fisheries, Indonesia.

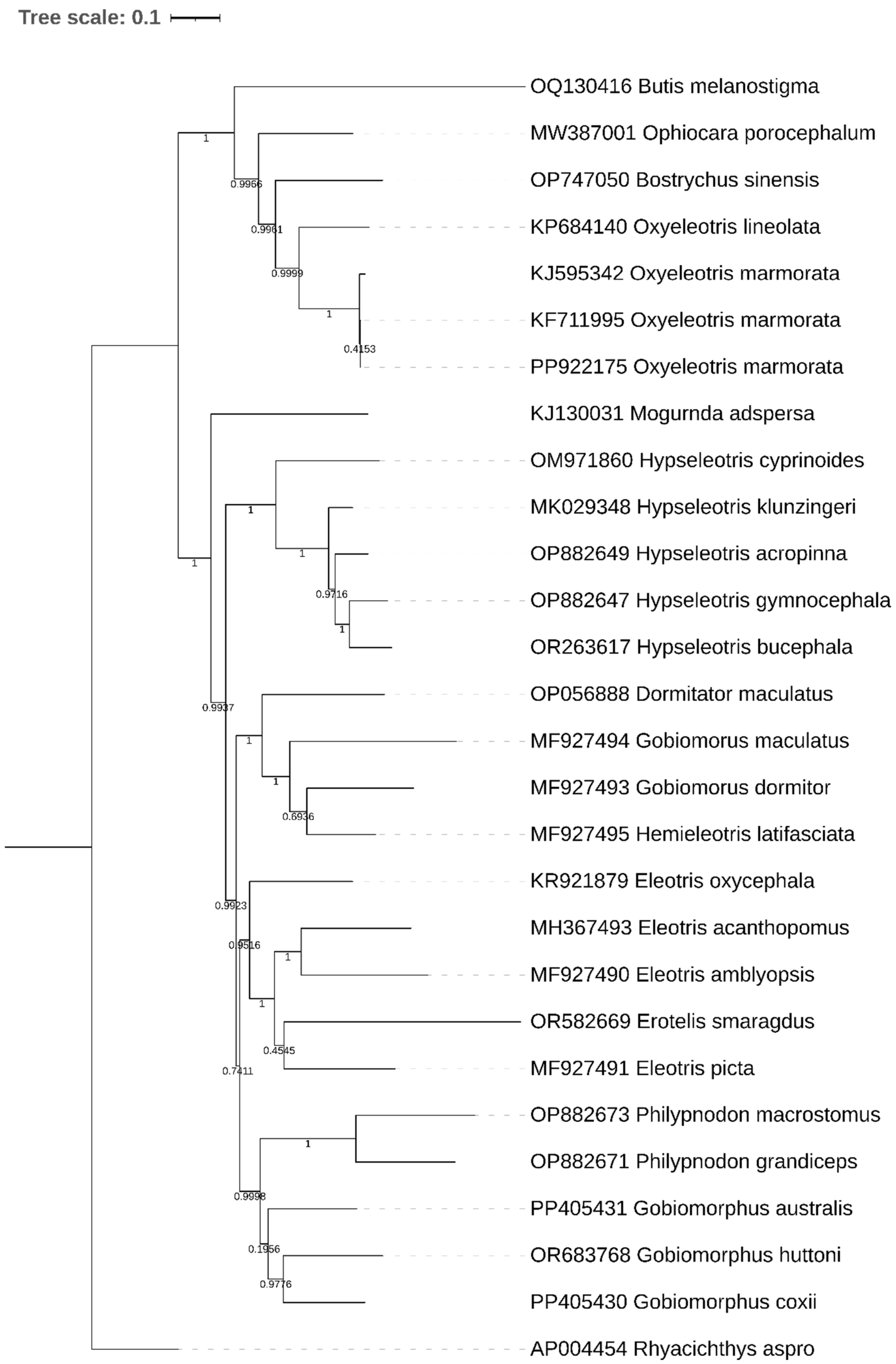

Figure S2. The maximum-likelihood phylogeny based on 13 concatenated PCGs clearly separates the newly sequenced *O. marmorata* from other Butidae and Eleotridae species.

Table S1. Details of the mitogenomes analyzed in this study, including the newly sequenced *O. marmorata* and additional Butidae and Eleotridae species retrieved from GenBank.

Sl. No.	Species	GenBank Accession Number	Locality Information	References
**Butidae**				
1	*Oxyeleotris marmorata*	PP922175	Lake Singkarak, West Sumatra, Indonesia	This study
2	*Oxyeleotris marmorata*	KF711995	Aquaculture Farm, Guangzhou, China	[1]
3	*Oxyeleotris marmorata*	KJ595342	Jinyang Breeding Farm, Guangzhou, China	[2]
4	*Oxyeleotris lineolata*	KP684140	Pearl River in Guangzou, China	[3]
5	*Butis melanostigma*	OQ130416	Taiwan	Unpublished
6	*Ophiocara porocephalum*	MW387001	Porong River, East Java, Indonesia	[4]
7	*Bostrychus sinensis*	OP747050	Coastal sea of China	[5]
**Eleotridae**				
8	*Mogurnda adspersa*	KJ130031	Southeastern Australia, Australia	[6]
9	*Hypseleotris acropinna*	OP882649	South Australia, Australia	Unpublished
10	*Hypseleotris bucephala*	OR263617	Queensland, Australia	Unpublished
11	*Hypseleotris cyprinoides*	OM971860	Guangzhou, China	[7]
12	*Hypseleotris gymnocephala*	OP882647	New South Wales, Australia	Unpublished
13	*Hypseleotris klunzingeri*	MK029348	Australia	[8]
14	*Dormitator maculatus*	OP056888	North Atlantic Ocean, USA	Unpublished
15	*Gobiomorus dormitor*	MF927493	Panama	Unpublished
16	*Gobiomorus maculatus*	MF927494	Panama	Unpublished
17	*Hemieleotris latifasciata*	MF927495	Río Tebario, Veraguas, Panama	[9]
18	*Eleotris acanthopomus*	MH367493	–	[10]
19	*Eleotris amblyopsis*	MF927490	Quebrada Jobito, Río Indio, Coclé, Panama	[9]
20	*Eleotris oxycephala*	KR921879	China	[11]
21	*Eleotris picta*	MF927491	Río Pichende, Río Piña, Darién, Panama	[9]
22	*Erotelis smaragdus*	OR582669	North Atlantic Ocean, USA	Unpublished
23	*Philypnodon grandiceps*	OP882671	South Australia, Australia	Unpublished
24	*Philypnodon macrostomus*	OP882673	South Australia, Australia	Unpublished
25	*Gobiomorphus australis*	PP405431	New South Wales, Australia	Unpublished
26	*Gobiomorphus coxii*	PP405430	New South Wales, Australia	Unpublished
27	*Gobiomorphus huttoni*	OR683768	North Atlantic Ocean, USA	Unpublished
28	*Rhyancichthys aspro*	AP004454	Outgroup	[12]

Relevant References

[1] Yang, Z.Y.; Liang, H.W.; Li, Z.; Wang, D.; Zou, G.W. Mitochondrial genome of the marbled goby (*Oxyeleotris marmorata*). *Mitochondrial DNA A DNA Mapp Seq Anal.* **2016**, *27*, 1073–1074.

[2] Xu, Y.; Hu, Y.; Bao, B.; Gong, X. Complete mitochondrial DNA sequence of marble goby, *Oxyeleotris marmorata* (Bleeker, 1852). *Mitochondrial DNA A DNA Mapp Seq Anal*. **2016**, *27*, 817–818.

[3] Chen, H.; Li, W.; Zhao, J.; Zhu, X. The complete mitochondrial genome of the sleepy cod (*Oxyeleotris lineolatus*). *Mitochondrial DNA A DNA Mapp Seq Anal*. **2016**, *27*, 2539–2540.

[4] Amin, M.H.F.; Lee, S.R.; Irawan, B.; Andriyono, S.; Kim, H.W. Characterization of the complete mitochondrial genome of the northern mud gudgeon, *Ophiocara porocephala* (Perciformes: Eleotridae) with phylogenetic implications. *Mitochondrial DNA B Resour*. **2021**, *6*, 953–955.

[5] Zhang, R.R.; Yang, K.; Luo, D.; Ding, S.X. A new species of *Bostrychus* (Gobiiformes: Eleotridae) from the East China Sea. *Zool. Res. Divers. Conserv.* **2024**, *1*, 82–85.

[6] da Rocha Perini, V.; de Carvalho, D.C.; Beheregaray, L.B.; Prosdocimi, F. The complete mitochondrial genome of the southern purple-spotted gudgeon *Mogurnda adspersa* (Perciformes: Eleotridae) through pyrosequencing. *Mitochondrial DNA A DNA Mapp Seq Anal*. **2016**, *27*, 380–382.

[7] Pan, Z.; Ruan, X.; Cheng, H.; Zhang, C.; Zhao, H. The complete mitochondrial genome of *Hypseleotris cyprinoides* (Perciformes: Eleotridae). *Mitochondrial DNA B Resour.* **2023**, *8*, 488–492.

[8] Schmidt, D.J.; McDougall, C. Complete mitogenomes of five ecologically diverse Australian freshwater fishes. *Mitochondrial DNA B Resour*. **2019**, *4*, 191–193.

[9] Alda, F.; Adams, A.J.; McMillan, W.O.; Chakrabarty, P. Complete mitochondrial genomes of three neotropical sleeper gobies: *Eleotris amblyopsis*, *E. picta*, and *Hemieleotris latifasciata* (Gobiiformes: Eleotridae). *Mitochondrial DNA B Resour*. **2017**, *2*, 747–750.

[10] Mennesson, M.I.; Bonillo, C.; Feunteun, E.; Keith, P. Phylogeography of *Eleotris fusca* (Teleostei: Gobioidei: Eleotridae) in the Indo-Pacific area reveals a cryptic species in the Indian Ocean. *Conserv. Genet.* **2018**, *19*, 1025–1038.

[11] Meng, Y.; Ma, H.; Ma, C.; Wei, H.; Liu, Y.; Zhang, F.; Wang, W.; Chen, W.; Zhao, M.; Chen, F.; Ma, L. The complete mitochondrial genome of *Eleotris oxycephala* (Perciformes: Eleotridae). *Mitochondrial DNA A DNA Mapp Seq Anal.* **2016**, *27*, 3820–3821.

[12] Miya, M.; Takeshima, H.; Endo, H.; Ishiguro, N.B.; Inoue, J.G.; Mukai, T.; Satoh, T.P.; Yamaguchi, M.; Kawaguchi, A.; Mabuchi, K.; Shirai, S.M.; Nishida, M. Major patterns of higher teleostean phylogenies: A new perspective based on 100 complete mitochondrial DNA sequences. *Mol. Phylogenet. Evol.* **2003**, *26*, 121–138.

Table S2. The intergenic nucleotides of different *Oxyeleotris* species.

Genes	*O. marmorata* (PP922175)	*O. marmorata* (KF711995)	*O. marmorata* (KJ595342)	*O. lineolata* (KP663727)	*O. lineolata* (KP684140)
tRNA-Phe (F)	0	0	0	0	0
12S rRNA	0	0	0	-5	4
tRNA-Val (V)	-1	2	2	-6	4
16S rRNA	1	0	0	0	14
tRNA-Leu (L2)	0	0	0	0	0
ND1	3	3	3	3	3
tRNA-Ile (I)	-1	-1	-1	-1	-1
tRNA-Gln (Q)	-1	-1	-1	-1	-1
tRNA-Met (M)	0	0	0	0	0
ND2	0	0	0	-3	0
tRNA-Trp (W)	3	3	3	3	3
tRNA-Ala (A)	1	1	1	1	1
tRNA-Asn (N)	37	37	37	37	37
tRNA-Cys (C)	0	0	0	-4	0
tRNA-Tyr (Y)	1	1	1	1	1
COI	0	0	0	0	11
tRNA-Ser (S2)	3	3	3	3	3
tRNA-Asp (D)	6	11	6	6	5
COII	0	0	0	0	0
tRNA-Lys (K)	1	1	1	1	1
ATP8	-10	-10	-10	-10	-10
ATP6	0	-1	-1	-1	-1
COIII	0	0	0	-1	0
tRNA-Gly (G)	0	0	0	0	0
ND3	0	0	0	-2	0
tRNA-Arg (R)	0	0	0	0	0
ND4L	-7	-7	-7	-7	0
ND4	0	0	0	0	0
tRNA-His (H)	0	0	0	0	0
tRNA-Ser (S1)	4	4	4	4	3
tRNA-Leu (L1)	0	0	0	0	0
ND5	-4	-4	-12	-12	-4
ND6	0	0	0	0	0
tRNA-Glu (E)	5	5	5	5	5
Cytb	0	0	0	0	0
tRNA-Thr (T)	-1	-1	-1	-1	-1
tRNA-Pro (P)	0	0	0	1	0
Control region	-	-	-	-	-

Table S3. The comparative frequencies of start and stop codons in 27 complete mitogenomes from 25 representative Butidae and Eleotridae species.

Genes	Start Codons	Nos. Sequences	Percentage	Stop Codons	Nos. Sequences	Percentage
ND1	ATG	27	100	TAG	15	55.56
				TAA	12	44.44
ND2	ATG	27	100	TA-	10	37.04
				T--	2	7.41
				TAA	15	55.56
COI	GTG	27	100	TAA	26	96.30
				T--	1	3.70
COII	ATG	27	100	T--	27	100.00
ATP8	ATG	27	100	TAA	26	96.30
				TAG	1	3.70
ATP6	ATG	27	100	TA-	4	14.81
				TAA	23	85.19
COIII	ATG	27	100	TA-	22	81.48
				T--	5	18.52
ND3	ATG	27	100	T--	27	100.00
ND4L	ATG	27	100	TAA	27	100.00
ND4	ATG	27	100	T--	27	100.00
ND5	ATG	27	100	TAA	24	88.89
				TAG	3	11.11
ND6	ATG	27	100	TAG	22	81.48
				TAA	5	18.52
Cyt b	ATG	27	100	T--	25	92.59
				TAA	1	3.70
				---	1	3.70

Table S4. The pairwise Ka/Ks ratios of 13 PCGs in representative Butidae and Eleotridae mitogenomes, including *O. marmorata*.

Genes	ND1	ND2	COI	COII	ATP8	ATP6	COIII	ND3	ND4L	ND4	ND5	ND6	Cytb
1	0	0.742857	0	0	0	0	0	0	0	0	0	0	0
2	0.143589744	1.241507	0.990712074	0	0.308880309	0	1.143790850	0	0	0.116959064	0.163043478	0.326360	0.080536913
3	0.068169067	0.123415	0.271971496	0.029515	0.159752705	0.079473	0.255046972	1.590840463	0.024126	0.081639804	0.123019571	0.223018	0.022208963
4	0.021545863	0.083048	0.323224044	0.008315	0.064841499	0.012901	0.243807403	1.404100529	0.039289	0.032220456	0.056080861	0.127148	0.026142402
5	0.028855599	0.085982	0.321146953	0.017661	0.077854261	0.031163	0.231333333	2.251861042	0.007058	0.036951833	0.073565847	0.122082	0.031963997
6	0.026233324	0.059366	0.285246796	0.006781	0.043769241	0.040087	0.263841633	4.267950963	0.022272	0.054912551	0.066298343	0.283535	0.031182917
7	0.040477602	0.087441	0.361451943	0.047420	0.150104239	0.043034	0.327841845	1.478186484	0.042196	0.049984477	0.071129707	0.251326	0.032241055
8	0.043750522	0.103220	0.321419888	0.04	0.144615752	0.051671	0.299908634	1.498995984	0.0304	0.050830647	0.069296182	0.258772	0.034536251
9	0.037471216	0.107259	0.341746593	0.050168	0.220141621	0.046139	0.351764706	1.723276723	0.020310	0.037167933	0.057260788	0.203569	0.035216526
10	0.095708988	0.147082	0.297285464	0.035855	0.167759616	0.082992	0.353052632	1.176513761	0.050319	0.071428571	0.109285409	0.303077	0.091434486
11	0.042004511	0.132402	0.330307263	0.045469	0.126569954	0.048269	0.342053199	2.013190955	0.036665	0.046006171	0.072079772	0.197671	0.031570881
12	0.042233040	0.128741	0.302182810	0.049063	0.116940582	0.039350	0.335521688	1.597722096	0.030053	0.045247975	0.064045710	0.294741	0.022555699
13	0.038186813	0.116007	0.393972603	0.039819	0.180854033	0.057914	0.396434848	1.904900817	0.036391	0.039119042	0.066970265	0.259970	0.029274171
14	0.038661754	0.131674	0.325543730	0.041393	0.113283296	0.054681	0.336085695	2.093768905	0.041636	0.064946203	0.074612053	0.237231	0.024747938
15	0.038436099	0.115085	0.285271638	0.053528	0.093120349	0.054920	0.336085695	3.243876464	0.038971	0.055328404	0.071989659	0.276069	0.037427354
16	0.055497304	0.096271	0.301772590	0.049209	0.193805470	0.066615	0.319770225	1.991510006	0.027597	0.070667681	0.099266205	0.275534	0.040224428
17	0.052318980	0.145065	0.296691792	0.053895	0.287874296	0.064572	0.295528455	1.908	0.048479	0.063510755	0.081025449	0.326399	0.044881176
18	0.040104561	0.165808	0.280810029	0.052283	0.213673382	0.054217	0.336103033	1.415943825	0.030837	0.059128967	0.074821946	0.347771	0.045736434
19	0.042376346	0.107601	0.310804802	0.049839	0.091615932	0.043160	0.397968606	2.558320373	0.018507	0.041645903	0.074687161	0.204222	0.029140219
20	0.038206421	0.092733	0.352272727	0.051987	0.070241473	0.039281	0.342796309	2.708836618	0.023294	0.044606211	0.078896974	0.226113	0.030792969
21	0.035210433	0.077250	0.3125	0.047296	0.072215721	0.057579	0.354277765	3.101980198	0.039361	0.049691225	0.073956154	0.163859	0.052993966
22	0.037021014	0.081255	0.262971958	0.054976	0.199074813	0.071459	0.291810180	1.534547152	0.028969	0.041162087	0.071713147	0.247432	0.058990908
23	0.046273781	0.096881	0.312672811	0.053707	0.133883884	0.070039	0.341959743	1.213273978	0.032799	0.072567716	0.063388071	0.306622	0.066651921
24	0.031494853	0.089542	0.333922261	0.028569	0.133012612	0.041573	0.325667981	1.892817680	0.021002	0.056741573	0.043409327	0.233976	0.024974836
25	0.034622836	0.077226	0.389059447	0.038898	0.056450764	0.053997	0.374650513	1.314155942	0.039155	0.035150432	0.071804541	0.365976	0.049492754
26	0.041982600	0.092026	0.325484040	0.054091	0.148837209	0.064724	0.393540373	2.025266704	0.021999	0.048839286	0.068786915	0.221829	0.038875243
**Average**	0.044632049	0.174105	0.331940221	0.038451	0.137275885	0.048839	0.345793935	1.842686064	0.028911	0.052555960	0.074632059	0.241704	0.038992093
**STDEV**	0.026029689	0.252195	0.151500283	0.017654	0.072752889	0.020913	0.180306643	0.889661775	0.013089	0.020981143	0.028063216	0.078595	0.019154348

Table S5. Amino acid abundance and RSCU values of PCGs among *Oxyeleotris* species.

***O. marmorata* (PP922175)**											
Codon	Count	RSCU	Codon	Count	RSCU	Codon	Count	RSCU	Codon	Count	RSCU
UUU(F)	97	1.06	UCU(S)	84	1.41	UAU(Y)	64	0.96	UGU(C)	17	0.67
UUC(F)	86	0.94	UCC(S)	96	1.61	UAC(Y)	69	1.04	UGC(C)	34	1.33
UUA(L)	87	0.98	UCA(S)	74	1.24	UAA(*)	55	1.05	UGA(*)	55	1.05
UUG(L)	52	0.58	UCG(S)	23	0.39	UAG(*)	47	0.90	UGG(W)	25	1.00
CUU(L)	104	1.17	CCU(P)	144	1.34	CAU(H)	59	0.86	CGU(R)	19	0.70
CUC(L)	130	1.46	CCC(P)	161	1.50	CAC(H)	79	1.14	CGC(R)	40	1.48
CUA(L)	119	1.33	CCA(P)	80	0.75	CAA(Q)	93	1.45	CGA(R)	28	1.04
CUG(L)	43	0.48	CCG(P)	44	0.41	CAG(Q)	35	0.55	CGG(R)	19	0.70
AUU(I)	96	1.14	ACU(T)	71	1.04	AAU(N)	82	1.01	AGU(S)	28	0.47
AUC(I)	79	0.94	ACC(T)	89	1.31	AAC(N)	81	0.99	AGC(S)	53	0.89
AUA(I)	77	0.92	ACA(T)	89	1.31	AAA(K)	81	1.54	AGA(R)	24	0.89
AUG(M)	40	1.00	ACG(T)	23	0.34	AAG(K)	24	0.46	AGG(R)	32	1.19
GUU(V)	30	1.03	GCU(A)	51	0.88	GAU(D)	30	0.78	GGU(G)	19	0.52
GUC(V)	29	1.00	GCC(A)	112	1.94	GAC(D)	47	1.22	GGC(G)	53	1.44
GUA(V)	43	1.48	GCA(A)	53	0.92	GAA(E)	51	1.07	GGA(G)	34	0.93
GUG(V)	14	0.48	GCG(A)	15	0.26	GAG(E)	44	0.93	GGG(G)	41	1.12
***O. marmorata* (KF711995)**											
Codon	Count	RSCU	Codon	Count	RSCU	Codon	Count	RSCU	Codon	Count	RSCU
UUU(F)	98	1.07	UCU(S)	84	1.40	UAU(Y)	64	0.96	UGU(C)	17	0.67
UUC(F)	86	0.93	UCC(S)	96	1.60	UAC(Y)	69	1.04	UGC(C)	34	1.33
UUA(L)	86	0.97	UCA(S)	74	1.24	UAA(*)	55	1.05	UGA(*)	55	1.05
UUG(L)	52	0.59	UCG(S)	23	0.38	UAG(*)	47	0.90	UGG(W)	25	1.00
CUU(L)	104	1.17	CCU(P)	144	1.34	CAU(H)	59	0.86	CGU(R)	19	0.70
CUC(L)	129	1.45	CCC(P)	162	1.51	CAC(H)	79	1.14	CGC(R)	40	1.48
CUA(L)	119	1.34	CCA(P)	80	0.74	CAA(Q)	93	1.45	CGA(R)	28	1.04
CUG(L)	43	0.48	CCG(P)	44	0.41	CAG(Q)	35	0.55	CGG(R)	19	0.70
AUU(I)	96	1.14	ACU(T)	71	1.04	AAU(N)	82	1.01	AGU(S)	28	0.47
AUC(I)	79	0.94	ACC(T)	90	1.32	AAC(N)	80	0.99	AGC(S)	54	0.90
AUA(I)	77	0.92	ACA(T)	88	1.29	AAA(K)	81	1.54	AGA(R)	24	0.89
AUG(M)	40	1.00	ACG(T)	24	0.35	AAG(K)	24	0.46	AGG(R)	32	1.19
GUU(V)	30	1.04	GCU(A)	51	0.88	GAU(D)	31	0.79	GGU(G)	19	0.52
GUC(V)	28	0.97	GCC(A)	112	1.94	GAC(D)	47	1.21	GGC(G)	53	1.44
GUA(V)	43	1.50	GCA(A)	53	0.92	GAA(E)	51	1.07	GGA(G)	34	0.93
GUG(V)	14	0.49	GCG(A)	15	0.26	GAG(E)	44	0.93	GGG(G)	41	1.12
***O. marmorata* (KJ595342)**											
Codon	Count	RSCU	Codon	Count	RSCU	Codon	Count	RSCU	Codon	Count	RSCU
UUU(F)	96	1.04	UCU(S)	84	1.40	UAU(Y)	63	0.94	UGU(C)	18	0.68
UUC(F)	88	0.96	UCC(S)	95	1.58	UAC(Y)	71	1.06	UGC(C)	35	1.32
UUA(L)	85	0.94	UCA(S)	75	1.25	UAA(*)	50	0.96	UGA(*)	74	1.42
UUG(L)	53	0.59	UCG(S)	27	0.45	UAG(*)	32	0.62	UGG(W)	23	1.00
CUU(L)	107	1.19	CCU(P)	144	1.33	CAU(H)	59	0.86	CGU(R)	19	0.70
CUC(L)	130	1.44	CCC(P)	162	1.50	CAC(H)	78	1.14	CGC(R)	39	1.43
CUA(L)	120	1.33	CCA(P)	80	0.74	CAA(Q)	94	1.49	CGA(R)	32	1.17
CUG(L)	46	0.51	CCG(P)	46	0.43	CAG(Q)	32	0.51	CGG(R)	28	1.02
AUU(I)	97	1.14	ACU(T)	68	1.01	AAU(N)	84	1.00	AGU(S)	28	0.47
AUC(I)	82	0.96	ACC(T)	89	1.32	AAC(N)	84	1.00	AGC(S)	51	0.85
AUA(I)	76	0.89	ACA(T)	88	1.30	AAA(K)	82	1.56	AGA(R)	20	0.73
AUG(M)	38	1.00	ACG(T)	25	0.37	AAG(K)	23	0.44	AGG(R)	26	0.95
GUU(V)	31	1.02	GCU(A)	52	0.91	GAU(D)	31	0.78	GGU(G)	21	0.58
GUC(V)	30	0.98	GCC(A)	108	1.89	GAC(D)	48	1.22	GGC(G)	51	1.42
GUA(V)	44	1.44	GCA(A)	54	0.94	GAA(E)	51	1.09	GGA(G)	30	0.83
GUG(V)	17	0.56	GCG(A)	15	0.26	GAG(E)	43	0.91	GGG(G)	42	1.17
***O. lineolata* (KP663727)**											
Codon	Count	RSCU	Codon	Count	RSCU	Codon	Count	RSCU	Codon	Count	RSCU
UUU(F)	98	1.07	UCU(S)	84	1.40	UAU(Y)	64	0.96	UGU(C)	17	0.67
UUC(F)	86	0.93	UCC(S)	96	1.60	UAC(Y)	69	1.04	UGC(C)	34	1.33
UUA(L)	86	0.97	UCA(S)	74	1.24	UAA(*)	55	1.05	UGA(*)	55	1.05
UUG(L)	52	0.59	UCG(S)	23	0.38	UAG(*)	47	0.90	UGG(W)	25	1.00
CUU(L)	104	1.17	CCU(P)	144	1.34	CAU(H)	59	0.86	CGU(R)	19	0.70

CUC(L)	129	1.45	CCC(P)	162	1.51	CAC(H)	79	1.14	CGC(R)	40	1.48
CUA(L)	119	1.34	CCA(P)	80	0.74	CAA(Q)	93	1.45	CGA(R)	28	1.04
CUG(L)	43	0.48	CCG(P)	44	0.41	CAG(Q)	35	0.55	CGG(R)	19	0.70
AUU(I)	96	1.14	ACU(T)	71	1.04	AAU(N)	82	1.01	AGU(S)	28	0.47
AUC(I)	79	0.94	ACC(T)	90	1.32	AAC(N)	80	0.99	AGC(S)	54	0.90
AUA(I)	77	0.92	ACA(T)	88	1.29	AAA(K)	81	1.54	AGA(R)	24	0.89
AUG(M)	40	1.00	ACG(T)	24	0.35	AAG(K)	24	0.46	AGG(R)	32	1.19
GUU(V)	30	1.04	GCU(A)	51	0.88	GAU(D)	31	0.79	GGU(G)	19	0.52
GUC(V)	28	0.97	GCC(A)	112	1.94	GAC(D)	47	1.21	GGC(G)	53	1.44
GUA(V)	43	1.50	GCA(A)	53	0.92	GAA(E)	51	1.07	GGA(G)	34	0.93
GUG(V)	14	0.49	GCG(A)	15	0.26	GAG(E)	44	0.93	GGG(G)	41	1.12
O. lineolata **(KP684140)**											
Codon	Count	RSCU	Codon	Count	RSCU	Codon	Count	RSCU	Codon	Count	RSCU
UUU(F)	98	1.05	UCU(S)	77	1.26	UAU(Y)	63	0.95	UGU(C)	25	0.76
UUC(F)	89	0.95	UCC(S)	95	1.56	UAC(Y)	70	1.05	UGC(C)	41	1.24
UUA(L)	91	1.03	UCA(S)	75	1.23	UAA(*)	62	1.18	UGA(*)	53	1.01
UUG(L)	51	0.58	UCG(S)	28	0.46	UAG(*)	43	0.82	UGG(W)	27	1.00
CUU(L)	116	1.32	CCU(P)	141	1.34	CAU(H)	56	0.88	CGU(R)	19	0.72
CUC(L)	107	1.21	CCC(P)	167	1.59	CAC(H)	71	1.12	CGC(R)	32	1.21
CUA(L)	117	1.33	CCA(P)	75	0.71	CAA(Q)	89	1.39	CGA(R)	31	1.17
CUG(L)	47	0.53	CCG(P)	37	0.35	CAG(Q)	39	0.61	CGG(R)	15	0.57
AUU(I)	92	1.12	ACU(T)	71	1.01	AAU(N)	78	0.91	AGU(S)	29	0.48
AUC(I)	86	1.05	ACC(T)	100	1.42	AAC(N)	93	1.09	AGC(S)	62	1.02
AUA(I)	68	0.83	ACA(T)	83	1.18	AAA(K)	79	1.50	AGA(R)	20	0.75
AUG(M)	41	1.00	ACG(T)	27	0.38	AAG(K)	26	0.50	AGG(R)	42	1.58
GUU(V)	32	1.07	GCU(A)	50	0.88	GAU(D)	22	0.70	GGU(G)	13	0.36
GUC(V)	29	0.97	GCC(A)	106	1.88	GAC(D)	41	1.30	GGC(G)	52	1.45
GUA(V)	42	1.40	GCA(A)	62	1.10	GAA(E)	62	1.29	GGA(G)	44	1.23
GUG(V)	17	0.57	GCG(A)	8	0.14	GAG(E)	34	0.71	GGG(G)	34	0.95

Table S6. RSCU values and CDsPT metrics for PCGs across *Oxyeleotris* species.

Amino Acids	***O. marmorata* (PP922175)**		***O. marmorata* (KF711995)**		***O. marmorata* (KJ595342)**		***O. lineolata* (KP663727)**		***O. lineolata* (KP684140)**	
	**Abundance**	**CDsPT**	**Abundance**	**CDsPT**	**Abundance**	**CDsPT**	**Abundance**	**CDsPT**	**Abundance**	**CDsPT**
Ala	231	60.8375	231	60.82148	229	60.04195	231	60.82148	226	59.59916
Arg	162	42.66526	162	42.65403	164	42.99948	162	42.65403	159	41.93038
Asn	163	42.92863	162	42.65403	168	44.04824	162	42.65403	171	45.09494
Asp	77	20.27917	78	20.53712	79	20.71316	78	20.53712	63	16.61392
Cys	51	13.43166	51	13.42812	53	13.89617	51	13.42812	66	17.40506
Gln	128	33.71082	128	33.70195	126	33.03618	128	33.70195	128	33.75527
Glu	95	25.01975	95	25.01316	94	24.64604	95	25.01316	96	25.31646
Gly	147	38.71477	147	38.70458	144	37.75564	147	38.70458	143	37.71097
His	138	36.34448	138	36.33491	137	35.92029	138	36.33491	127	33.49156
Ile	252	66.36819	252	66.35071	255	66.85894	252	66.35071	246	64.87342
Leu	535	140.9007	533	140.3370	541	141.8458	533	140.3370	529	139.5042
Lys	105	27.65341	105	27.64613	105	27.53015	105	27.64613	105	27.68987
Met	40	10.53463	40	10.53186	38	9.963293	40	10.53186	41	10.81224
Phe	183	48.19594	184	48.44655	184	48.24331	184	48.44655	187	49.31435
Pro	429	112.9839	430	113.2175	432	113.2669	430	113.2175	420	110.7595
Ser	358	94.28496	359	94.52343	360	94.38909	359	94.52343	366	96.51899
Thr	272	71.63550	273	71.87994	270	70.79182	273	71.87994	281	74.10338
Trp	25	6.584145	25	6.582412	23	6.030414	25	6.582412	27	7.120253
Tyr	133	35.02765	133	35.01843	134	35.13372	133	35.01843	133	35.07384
Val	116	30.55043	115	30.27909	122	31.98741	115	30.27909	120	31.64557
Stp	157	41.34843	157	41.33755	156	40.90194	157	41.33755	158	41.66667

Table S7. Detailed comparison of anticodon sequences in tRNA genes among the five *Oxyeleotris* mitogenomes.

Genes	***O. marmorata* (PP922175)**	***O. marmorata* (KF711995)**	***O. marmorata* (KJ595342)**	***O. lineolata* (KP663727)**	***O. lineolata* (KP684140)**
tRNA-Phe (F)	GAA	GAA	GAA	GAA	GAA
tRNA-Val (V)	TAC	TAC	TAC	TAC	TAC
tRNA-Leu (L2)	TAA	TAA	TAA	TAA	TAA
tRNA-Ile (I)	GAT	GAT	GAT	GAT	GAT
tRNA-Gln (Q)	TTG	TTG	TTG	TTG	TTG
tRNA-Met (M)	CAT	CAT	CAT	CAT	CAT
tRNA-Trp (W)	TCA	TCA	---	TCA	TCA
tRNA-Ala (A)	TGC	TGC	TGC	TGC	TGC
tRNA-Asn (N)	GTT	GTT	GTT	GTT	GTT
tRNA-Cys (C)	GCA	GCA	GCA	GCA	GCA
tRNA-Tyr (Y)	GTA	GTA	GTA	GTA	GTA
tRNA-Ser (S2)	TGA	TGA	TGA	TGA	TGA
tRNA-Asp (D)	GTC	GTC	GTC	GTC	GTC
tRNA-Lys (K)	TTT	TTT	TTT	TTT	TTT
tRNA-Gly (G)	TCC	TCC	TCC	TCC	TCC
tRNA-Arg (R)	TCG	TCG	TCG	TCG	TCG
tRNA-His (H)	GTG	GTG	GTG	GTG	GTG
tRNA-Ser (S1)	GCT	GCT	GCT	GCT	GCT
tRNA-Leu (L1)	TAG	TAG	TAG	TAG	TAG
tRNA-Glu (E)	TTC	TTC	TTC	TTC	TTC
tRNA-Thr (T)	TGT	TGT	TGT	TGT	TGT
tRNA-Pro (P)	TGG	TGG	TGG	TGG	TGG

Table S8. The haplotype network of *O. marmorata* from native and introduced habitats.

**Species**	**GenBank Accession Number**	**Locality Information**	**Haplotype**
O. marmorata	PP922175	Sumatra (Indonesia)	Hap_1 [1]
O. marmorata	KF711995	Guangzhou (China)	Hap_2 [1]
O. marmorata	KJ595342	Guangzhou (China)	Hap_3 [1]

Table S9. The comparison of genetic distances for *O. marmorata* between native and introduced habitats, along with analysis of variable nucleotide and amino acid sites between two *Oxyeleotris* species.

Genes	Genetic Distance	Nucleotide	Amino Acid
ND1	0.007	149	15
ND2	0.016	160	73
COI	0.032	223	97
COII	0.003	59	6
ATP8	0.012	18	4
ATP6	0.003	96	14
COIII	0.017	95	37
ND3	0.003	87	10
ND4L	0.000	75	15
ND4	0.006	194	32
ND5	0.007	234	51
ND6	0.012	72	19
Cytb	0.004	166	16
